# Single-Machine Scheduling to Minimize Total Completion Time and Tardiness with Two Competing Agents

**DOI:** 10.1155/2014/596306

**Published:** 2014-01-19

**Authors:** Wen-Chiung Lee, Yau-Ren Shiau, Yu-Hsiang Chung, Lawson Ding

**Affiliations:** ^1^Department of Statistics, Feng Chia University, Taichung, Taiwan; ^2^Department of Industrial Engineering and System Management, Feng Chia University, Taichung, Taiwan; ^3^Department of Industrial & Engineering Management, National Chiao Tung University, Hsinchu, Taiwan

## Abstract

We consider a single-machine two-agent problem where the objective is to minimize a weighted combination of the total completion time and the total tardiness of jobs from the first agent given that no tardy jobs are allowed for the second agent. A branch-and-bound algorithm is developed to derive the optimal sequence and two simulated annealing heuristic algorithms are proposed to search for the near-optimal solutions. Computational experiments are also conducted to evaluate the proposed branch-and-bound and simulated annealing algorithms.

## 1. Introduction

In traditional scheduling, there is a common goal to minimize for all the jobs [1–4]. In contrast to that, there is a growing interest in multiagent scheduling problems where jobs are from several customers who have different goals to pursue. For instance, Peha [5] gave the telecommunication service examples where various types of packets and service compete for the use of a commercial satellite, and the problem is to satisfy the service requirements of individual agents to transfer voice, image, and text files for their clients. Brewer and Plott [6] gave a transportation example where the agents own transportation resources and compete for the usage of the infrastructures. Kubzin and Strusevich [7] presented an example that maintenance operations complete with the real jobs for machine occupancy on maintenance planning. Baker and Smith [8] gave a sharing example of a prototype shop in which the department of research and development might be concerned about quick response time, while the department of manufacturing might be more concerned about meeting the due dates.

Agnetis et al. [9] and Baker and Smith [8] were the pioneers that brought the multiagent problems into scheduling field. Agnetis et al. [9] considered the maximum of regular functions, number of late jobs, and total weighted completion times. They obtained different scenarios depending on the objective function of each agent and on the structure of the processing system. For each scenario, they addressed the complexity of various problems. Baker and Smith [8] examined the implications of minimizing an aggregate scheduling objective function in which jobs belonging to different customers are evaluated based on their individual criteria. They demonstrated that the problem to minimize a mix of makespan, maximum lateness, or total weighted completion time is NP-hard. Cheng et al. [10] considered the feasibility model of multiagent scheduling on a single machine where each agent's objective function is to minimize the total weighted number of tardy jobs. They showed that the general problem is strongly NP-complete and developed the complexity results for some special cases. Ng et al. [11] addressed a single-machine two-agent problem to minimize the total completion time of the first agent given that the number of tardy jobs of the second agent cannot exceed a certain number. They showed that the problem is NP-hard under high multiplicity encoding and can be solved in pseudopolynomial time under binary encoding. Lee et al. [12] discussed a single-machine multiagent scheduling problem in which each agent is responsible for his own set of jobs and wishes to minimize the total weighted completion time of his own set of jobs. They reduced this NP-hard problem to a multiobjective short-path problem. They also provided an efficient approximation algorithm with a reasonably good worst-case ratio. Agnetis et al. [13] developed the branch-and-bound algorithms for several single-machine two-agent scheduling problems. They used Lagrangian dual to derive the bounds for the branch-and-bound algorithm in strongly polynomial time. Lee et al. [14] studied a single-machine two-agent problem with deteriorating jobs in which the objective function is to minimize the total completion time of jobs from one agent given that no tardy jobs are allowed for the other agent. They provided a branch-and-bound algorithm to derive the optimal solution and several heuristic algorithms for the proposed problem. Recently, Leung et al. [15] generalized the single-machine problems proposed by Agnetis et al. [9] to the case of multiple identical parallel machines. In addition, they also considered the situations where the jobs may have different release dates and preemptions may or may not be allowed. Nong et al. [16] studied a single-machine two-agent scheduling problem for minimizing the total cost in which the cost of the first agent is the maximum weighted completion time while that of the second agent is the total weighted completion time. Liu et al. [17] considered single-machine two-agent problems with increasing linear deterioration consideration. They developed the optimal solutions for some problems where the goal is to minimize the objective function of the first agent given that the objective function of the second agent cannot exceed a certain upper bound. Cheng et al. [18] studied a two-agent single-machine scheduling problem with release times where the objective is to minimize the total weighted completion time of the jobs of one agent with the constraint that the maximum lateness of the jobs of the other agent does not exceed a given limit. They proposed a branch-and-bound algorithm to solve problems with up to 24 jobs. Yu et al. [19] developed the optimal solutions for several single-machine scheduling problems with two competing agents and maintenance activity. Liu et al. [20] considered single-machine scheduling with two-agent and sum-of-processing-times-based deterioration effect. They developed some polynomial-time algorithms for the respective problems.

Pinedo [1] pointed out that a customer is often concerned with multiple objectives. For instance, he might want to have a lower cost and the on-time delivery. To the best of our knowledge, multiagent scheduling problems with the consideration of multiple objectives from the same agent have seldom been discussed in the literature. In this paper, we study a two-agent scheduling problem on a single machine where the objective is to minimize the weighted combination of the total completion time and the total tardiness of jobs from the first agent given that the number of tardy jobs from the second agent is zero. The rest of the paper is organized as follows. In the next section the formulation of our problem is described. In Section 3, a branch-and-bound algorithm incorporating several elimination rules and a lower bound is constructed to speed up the search for the optimal solution. In Section 4, two simulated annealing algorithms are proposed to solve this problem. In Section 5, a computational experiment is conducted to evaluate the efficiency of the branch-and-bound algorithm and the performance of the proposed simulated annealing algorithms. A conclusion is given in the final section.

## 2. Problem Description

The problem we study is described as follows. There are *n* jobs ready to be processed. Each job belongs to either one of the two agents AG_1_ or AG_2_. For each job *j*, there is a processing time *p*_*j*_, a due date *d*_*j*_, and an agent code *I*_*j*_, where *I*_*j*_ = 1 if job *j* ∈ AG_1_ or *I*_*j*_ = 2 if job *j* ∈ AG_2_. Under a schedule *S*, let *C*_*j*_(*S*) be the completion time of job *j*, let *T*_*j*_(*S*) = max{0, *C*_*j*_(*S*) − *d*_*j*_} be the tardiness of job *j*, and *U*_*j*_(*S*) = 1 if *T*_*j*_(*S*) > 0 and zero otherwise. In this paper, we study a single machine problem to minimize the weighted combination of the total completion time and the total tardiness of jobs from AG_1_ given that no tardy jobs from AG_2_ are allowed. Using the conventional three fields of notation, this problem is denoted by 1 | ∑_*j*∈AG_2__ *U*_*j*_ = 0|∑_*j*∈AG_1__ *θT*_*j*_ + (1 − *θ*)*C*_*j*_ where 0 ≤ *θ* ≤ 1.

## 3. A Branch-and-Bound Algorithm

If there is no job from agent AG_2_ and *θ* = 1, the problem under consideration reduces to the classical single-machine total tardiness time problem which is proved to be NP-hard by Du and Leung [21]. Therefore, a branch-and-bound algorithm might be a good way to derive the optimal solution. In this section, we will provide several dominance properties to speed up the search process.

### 3.1. Dominance Properties

In this subsection, we develop a nonadjacent and several adjacent dominance properties to reduce the searching scope.

**Property 1**

If jobs *i*, *j* ∈ AG_1_, *d*_*i*_ ≤ *d*_*j*_, and *p*_*i*_ < *p*_*j*_, then job *i* must precede job *j* in an optimal schedule.

The following propositions are the ordering criteria on a pair of adjacent jobs. Suppose that *S* and *S*′ are two schedules of jobs and the only difference between them is a pairwise interchange of two adjacent jobs *i* and *j*. That is, *S* = (*π*, *i*, *j*, *π*′) and *S*′ = (*π*, *j*, *i*, *π*′), where *π* and *π*′ each denote a partial sequence. In addition, let *t* denote the completion time of the last job in *π*. The completion times of jobs *i* and *j* in *S* are

(1) $$\begin{array}{c} C_{i}\left(S\right)=t+p_{i}, \end{array}$$

(2) $$\begin{array}{c} C_{j}\left(S\right)=t+p_{i}+p_{j}. \end{array}$$

Similarly, the completion times of jobs *j* and *i* in *S*′ are

(3) $$\begin{array}{c} C_{j}\left(S^{\prime }\right)=t+p_{j}, \end{array}$$

(4) $$\begin{array}{c} C_{i}\left(S^{\prime }\right)=t+p_{j}+p_{i}. \end{array}$$

**Proposition 1**

If jobs *i*, *j* ∈ *AG*_1_, *p*_*i*_ < *p*_*j*_, and *d*_*j*_ < *d*_*i*_ < *t* + *p*_*j*_, then *S* dominates *S*′.

**Proof**

Since jobs in partial sequence *π* are processed in the same order in both *S* and *S*′ and from (2) and (4), we have

(5) $$\begin{array}{c} C_{k}\left(S\right)=C_{k}\left(S^{\prime }\right)\;\text{if}\;\text{job}\;k\in \pi_{}\;\text{o}{\text{r}}_{}\;\;\pi^{\prime }. \end{array}$$

To show that *S* dominates *S*′, it suffices to show

(6) θ[Ti(S)+Tj(S)]+(1−θ)[Ci(S)+Cj(S)] <θ[Ti(S′)+Tj(S′)]+(1−θ)[Ci(S′)+Cj(S′)].

Since *p*_*i*_ < *p*_*j*_ and *d*_*j*_ < *d*_*i*_ < *t* + *p*_*j*_, we have

(7) $$\begin{array}{c} T_{i}\left(S\right)=\max\left\{t+p_{i}-d_{i},0\right\},\\ T_{j}\left(S\right)=t+p_{i}+p_{j}-d_{j},\\ T_{j}\left(S^{\prime }\right)=t+p_{j}-d_{j},\\ T_{i}\left(S^{\prime }\right)=t+p_{j}+p_{i}-d_{i}. \end{array}$$

Suppose that *T*_*i*_(*S*) is not zero. Note that this is the more restrictive case since it comprises the case that *T*_*i*_(*S*) is zero. From *p*_*i*_ < *p*_*j*_, we have

(8) θ[Ti(S)+Tj(S)]+(1−θ)[Ci(S)+Cj(S)]  −θ[Ti(S′)+Tj(S′)]−(1−θ)[Ci(S′)+Cj(S′)] =pi−pj<0.

Thus, *S* dominates *S*′.

**Proposition 2**

If jobs *i*, *j* ∈ *AG*_1_, (*p*_*i*_ − *p*_*j*_) + *θ*(*d*_*i*_ − *t* − *p*_*i*_) < 0, *d*_*j*_ < *t* + *p*_*j*_ < *d*_*i*_ < *t* + *p*_*i*_ + *p*_*j*_, and *p*_*i*_ < *p*_*j*_, then *S* dominates *S*′.

**Proposition 3**

If jobs *i*, *j* ∈ *AG*_1_, *d*_*j*_ < *t* + *p*_*j*_, *t* + *p*_*i*_ + *p*_*j*_ < *d*_*i*_, and *p*_*i*_ − (1 − *θ*)*p*_*j*_ < 0, then *S* dominates *S*′.

**Proposition 4**

If jobs *i*, *j* ∈ *AG*_1_, *t* + *p*_*i*_ < *d*_*i*_, *t* + *p*_*j*_ < *d*_*j*_, *t* + *p*_*i*_ + *p*_*j*_ > max{*d*_*i*_, *d*_*j*_}, and *θ*(*d*_*i*_ − *d*_*j*_) + (1 − *θ*)(*p*_*i*_ − *p*_*j*_) < 0, then *S* dominates *S*′.

**Proposition 5**

If jobs *i*, *j* ∈ *AG*_1_, *t* + *p*_*j*_ < *d*_*j*_ < *t* + *p*_*i*_ + *p*_*j*_ < *d*_*i*_, and *θ*(*t* + 2*p*_*j*_ − *d*_*j*_) + (*p*_*i*_ − *p*_*j*_) < 0, then *S* dominates *S*′.

**Proposition 6**

If jobs *i*, *j* ∈ *AG*_1_, *p*_*i*_ < *p*_*j*_, and *t* + *p*_*i*_ + *p*_*j*_ < *d*_*j*_ < *d*_*i*_, then *S* dominates *S*′.

**Proposition 7**

If jobs *i*, *j* ∈ *AG*_1_, *t* + *p*_*j*_ < *d*_*j*_ < *t* + *p*_*i*_ + *p*_*j*_, (*p*_*i*_ − *p*_*j*_) + *θ*(*t* + *p*_*j*_ − *d*_*j*_) < 0, and *d*_*i*_ < *t* + *p*_*i*_, then *S* dominates *S*′.

**Proposition 8**

If jobs *i*, *j* ∈ *AG*_1_, *d*_*i*_ < *t* + *p*_*i*_, *t* + *p*_*i*_ + *p*_*j*_ < *d*_*j*_, and (1 − *θ*)*p*_*i*_ < *p*_*j*_, then *S* dominates *S*′.

**Proposition 9**

If job *i* ∈ *AG*_1_, job *j* ∈ *AG*_2_, *p*_*i*_ < *θp*_*j*_, and *t* + *p*_*i*_ + *p*_*j*_ < *d*_*j*_, then *S* dominates *S*′.

To further facilitate the search process, we provide two properties to determine the feasibility of a partial schedule or the ordering of the remaining unscheduled jobs. Assume that (*π*, *π* ^*c*^) is a sequence of jobs where *π* is the scheduled part with *k* jobs and *π* ^*c*^ is the unscheduled part with (*n* − *k*) jobs. Among the unscheduled jobs, there are *n*_1_ jobs from agent AG_1_ and *n*_2_ jobs from agent AG_2_ where *n*_1_ + *n*_2_ = *n* − *k*. Let S* = (*π*, π*) be a sequence such that *n*_1_ jobs from agent AG_1_ are first scheduled in the shortest processing time (SPT) rule, followed by *n*_2_ jobs from agent AG_2_ in the earliest due date (EDD) rule. In addition, let *d*_(1)_^2^ ≤ *d*_(2)_^2^ ≤ ⋯≤*d*_(*n*_2_)_^2^ denote the due dates of the remaining *n*_2_ jobs from agent AG_2_ when they are arranged in the EDD rule. Moreover, let *p*_(1)_^2^, *p*_(2)_^2^,…, *p*_(*n*_2_)_^2^ denote their corresponding processing times and let *C*_[*k*]_ be the completion times of the last job in *π*.

**Property 2**

If there exists a *j* such that *C*_[*k*]_ + ∑_*i*=1_^*j*^ *p*_(*i*)_^2^ − *d*_(*j*)_^2^ > 0 for some *j* = 1, 2, …, *n*_2_, then sequence (*π*, *π* ^*c*^) is infeasible.

**Property 3**

If ∑_*k*∈π*_ *U*_*k*_(S*) = 0, then S* = (*π*, π*) dominates sequences of the type (*π*, *π* ^*c*^).

### 3.2. A Lower Bound

The efficiency of the branch-and-bound algorithm also depends on the lower bound of the partial sequence. Assume that PS is a partial schedule in which the order of the first *k* jobs is determined and let US be the unscheduled part with (*n* − *k*) jobs. Among the unscheduled jobs, there are *n*_1_ jobs from agent AG_1_ and *n*_2_ jobs from agent AG_2_. Moreover, let *d*_(1)_^2^ ≤ *d*_(2)_^2^ ≤ ⋯≤*d*_(*n*_2_)_^2^ denote the due dates of the remaining *n*_2_ jobs from agent AG_2_ when they are arranged in the EDD rule and *p*_(1)_^2^, *p*_(2)_^2^,…, *p*_(*n*_2_)_^2^ denote their corresponding processing times. In addition, let *p*_(1)_^1^ ≤ *p*_(2)_^1^ ≤ ⋯≤*p*_(*n*_1_)_^1^ denote the processing times of the remaining *n*_1_ jobs from agent AG_1_ when they are arranged in the SPT rule and *d*_(1)_^1^ ≤ *d*_(2)_^1^ ≤ ⋯≤*d*_(*n*_1_)_^1^ denote the due dates of the remaining *n*_1_ jobs from agent AG_1_ when they are arranged in the EDD rule. To derive the lower bound of the objective function, we construct *n*_1_ pseudo jobs from agent AG_1_ in the way that job *j* from agent AG_1_ has the processing time *p*_(*j*)_^1^ and due date *d*_(*j*)_^1^. The idea to derive the lower bound of the completion times of jobs from agent AG_1_ is to assign the completion times to jobs from agent AG_2_ as late as possible without violating the assumption of no tardy jobs from agent AG_2_ and then to proceed jobs from agent AG_1_ into the machine available periods with the assumption that jobs splitting is allowed for jobs from agent AG_1_. In addition, let *t* be the completion time of the last job in partial schedule PS. The procedures are basically divided into two phases. The first phase is to calculate the latest time to start processing jobs from AG_2_, and the second phase is to derive the lower bounds for the completion times of jobs from agent AG_1_. The steps are given as follows.

*Phase I*

 
*Step  1.* Set *i* = *n*_2_, *st* = *∞*, and *t*_*n*_2_+1_ = *∞*.
 
*Step  2.* If *d*_(*i*)_^2^ < *st*, set *t*_*i*_ = *d*_(*i*)_^2^ − *p*_(*i*)_^2^. Otherwise, set *t*_*i*_ = *st* − *p*_(*i*)_^2^.
 
*Step  3.* Set *st* = *t*_*i*_ and *i* = *i* − 1. If *i* ≥ 1, go to Step  2.
 
*Step  4. *Output (*t*_1_, *t*_2_,…, *t*_*n*_2__, *t*_*n*_2_+1_).

*Phase II*

 
*Step  1. *Set *i* = 1 and *k* = 0.
 
*Step  2*. Set *k* = *k* + 1.
 
*Step  3. *If *t* + *p*_(*i*)_^1^ > *t*_*k*_, set *p*_(*i*)_^1^ = *p*_(*i*)_^1^ − (*t*_*k*_ − *t*), *t* = *t*_*k*_ + *p*_(*k*)_^2^ and go to Step  2. Otherwise, set *t* = *t* + *p*_(*i*)_^1^ and *C*_(*i*)_^1^ = *t*.
 
*Step  4. *If *i* < *n*_1_, set *i* = *i* + 1 and go to Step  3.
 
*Step  5.* Output (*C*_(1)_^1^, *C*_(2)_^1^,…, *C*_(*n*_1_)_^1^).

Thus, the lower bound of the weighted combination of the total completion time and the total tardiness for PS is

(9) LB(PS)=∑j∈AG1∩PS((1−θ)C[j](PS)+θT[j](PS)) +∑j=1n1(1−θ)C(j)1+θmax{0,C(j)1−d(j)1}.

## 4. The Simulated Annealing Algorithm

Metaheuristic algorithms have been successfully applied to solve many scheduling or optimization problems [22–25]. The simulated annealing (SA) algorithm is one of the most popular ones. It is originally proposed by Kirkpatrick et al. [26] and has been successfully applied to solve many combinatorial optimization problems. The advantage of SA algorithm is that it could avoid getting trapped in a local optimum. In this paper, we utilize the SA algorithm to derive the near-optimal solution for the proposed problem. A brief description of the SA procedure is as follows. Given an initial sequence, a new sequence is created by a random neighborhood generation. The new sequence is accepted if it has a smaller objective function than the original sequence; otherwise, it is accepted with some probability that decreases as the process evolves. The exchange condition is initially set to a high level so that a neighborhood exchange could happen frequently in early iterations. It is gradually lowered using a predetermined cooling strategy so that it becomes more difficult to exchange in later iterations unless a better solution is obtained.

The key elements of the SA algorithm are as follows.

(1) *Initial Sequence.* Since no tardy job is allowed for agent AG_2_ and the SPT rule yields the optimal solution for the total completion time problem, the initial sequence is constructed as follows. Jobs from agent AG_2_ are first placed according to the EDD rule, followed by jobs from agent AG_1_ according to the SPT rule.

(2) *Neighborhood Generation.* Neighborhood generation plays an important role in the efficiency of the SA method. Three neighborhood generation methods are used in the preliminary study. They are the pairwise interchange (PI), the extraction and forward-shifted reinsertion (EFSR), and the extraction and backward-shifted reinsertion (EBSR) movements. It is observed that the PI movement yields a better solution in general. Thus, it is used in later analysis.

(3) *Acceptance Probability.* In SA, solutions are accepted according to the magnitude of increase in the objective function and the temperature. The probability of acceptance is generated from an exponential distribution:

(10) $$\begin{array}{c} P\left(\text{accept}\right)=\exp\left(-\alpha \times \Delta \text{TC}\right), \end{array}$$

where *α* is the control parameter and ΔTC is the change in the objective function. In addition, the method of changing *α* at the *k*th iteration is obtained from Ben-Arieh and Maimon [27] and is given by

(11) $$\begin{array}{c} \alpha =\frac{k}{\beta }, \end{array}$$

where *β* is an experimental factor. After some pretests, we chose *β* = 6000. If the weighted combination of the total completion time and the total tardiness increases as a result of a random neighborhood movement, the new sequence is accepted when *P*(accept) > *r*, where *r* is a uniform random number between 0 and 1.

(4) *Objective Function.* The objective function is usually chosen to be the one that we want to minimize. Thus, in the first simulated annealing algorithm SA_1_, the objective function is ∑_*j*∈AG_1__ *θT*_*j*_ + (1 − *θ*)*C*_*j*_ and regenerates the neighborhood if it is infeasible. Since our problem is to minimize an objective function under a constraint, it is reasonable to add the constraint into the objective function. Thus, in the second simulated annealing algorithm SA_2_, the objective function is modified to ∑_*j*∈AG_1__ *θT*_*j*_ + (1 − *θ*)*C*_*j*_ + *λ*∑_*j*∈AG_2__ *T*_*j*_ where *λ* is chosen to be 1000 after some preliminary tests.

(5) *Stopping Condition.* Our preliminary tests showed that the schedule is quite stable after 400*n* iterations, where *n* is the number of jobs. Thus, 400*n* was used as the number of iterations.

## 5. Computational Experiments

In this section, we conducted the computational experiments to evaluate the performance of the branch-and-bound and the proposed SA algorithms. The algorithms were coded in Fortran 90 and run on a personal computer with Intel(R) Core(TM)2 Duo CPU T7500 2.20 GHz and 2.99 GB RAM under Windows XP. The job processing times were generated from a uniform distribution over the integers 1 to 100 and the due dates of jobs were generated from a uniform distribution over the integers between *T*(1 − *τ* − *R*/2) and *T*(1 − *τ* + *R*/2), where *R* is the due date range factor, *τ* is the tardiness factor, and *T* is the total processing time of all the jobs.

The computational experiments were divided into four parts. In the first part of the experiment, we studied the effects of the due date factors *τ* and *R* and the proportion of jobs from the second agent *P* to the performance of the branch-and-bound algorithm. The job size *n* was 12, and the coefficient of the weighted combination of the total completion time and the total tardiness *θ* was 0.5. Three values of the proportion of jobs from agent AG_1_ were considered, that is, *P* = 0.25, 0.5, and 0.75. Eight combinations of (*τ*, *R*) values were tested, that is, (0.25, 0.25), (0.25, 0.50), (0.25, 0.75), (0.5, 0.25), (0.5, 0.50), (0.5, 0.75), (0.75, 0.25), and (0.75, 0.50). The mean and the standard deviation of the number of nodes and the mean and the standard deviation of the CPU time (in seconds) were reported for the branch-and-bound algorithm. 100 replications were randomly generated for each case and the results were presented in Table 1. A two-way analysis of variance (ANOVA) on the number of nodes of the branch-and-bound algorithm was constructed and given in Table 2. The resulting *F* value of factor *P* was 189.52 with a *P* value close to 0, which indicated that the values of *P* really affect the performance of the branch-and-bound algorithm. In fact, problems are easier to solve when the value of *P* is larger. The main reason is that Property 2 is more powerful in this case. In addition, the resulting *F* value of due date factor *τ* or *R* was 29.19 with a *P* value close to 0, which indicated that the values of *τ* or *R* have statistically significant effects on the difficulty of the problem. A closer look at Table 1 revealed that problems are easier to solve as *R* decreases or when *τ* = 0.75. The main reason is that Property 2 and the lower bound are more powerful in those cases.

Similar to the first part of the experiment, the second part was to test the effects of the coefficient *θ* as well as the effects of the due date factors *τ* and *R*. The job size was fixed at 12, and *P* was 0.5. Three different values of *θ* (0.25, 0.5, and 0.75) and eight values of (*τ*, *R*) values were tested, that is, (0.25, 0.25), (0.25, 0.50), (0.25, 0.75), (0.5, 0.25), (0.5, 0.50), (0.5, 0.75), (0.75, 0.25), and (0.75, 0.50). As a consequence, 24 cases were tested and 100 replications were randomly generated for each case. The results were presented in Table 3. A two-way ANOVA on the number of nodes was utilized to test the effects of the parameters to the performance of the branch-and-bound algorithm, and the result was reported in Table 4. The resulting *F* value for the coefficient *θ* was 0.74 with a *P* value of 0.48, which implied that *θ* does not affect the performance of the branch-and-bound algorithm. On the other hand, the statistical test showed that the due date factors have significant effects on the performance of the branch-and-bound algorithm, and the problems are easier to solve when *τ* = 0.75, which is consistent with the findings in the first part of the experiment.

The main purpose of the third part of the experiment was to study the impact of the number of jobs to the performance of the branch-and-bound algorithms and the accuracy of the proposed simulated annealing algorithms. Three different job sizes (*n* = 16, 20, and 24) were tested. Since the problems are relatively easier to solve when *τ* = 0.75 and *θ* was found to be the insignificant factors in the second part of the experiments, we ignored the cases when *τ* = 0.75 and fixed *θ* at 0.5 in the third part of the experiment. Two values of *τ* (0.25, 0.50) and three values of *P* (0.25, 0.50, and 0.75) and of *R* (0.25, 0.50, and 0.75) were tested. The mean and standard deviation of the number of nodes and the mean and standard deviation of the CPU time (in seconds) for the branch-and-bound algorithm were given, while only the mean and standard deviation of the error percentages of the SA algorithms were reported. The error percentage of the solution produced by SA is calculated as

(12) $$\begin{array}{c} \frac{\left(V-V^{*}\right)}{V^{*}}\times 100\%, \end{array}$$

where *V* is the weighted combination of the total completion time and the total tardiness of the solution generated by SA and V* is the value of the optimal solution obtained from the branch-and-bound algorithm. The execution time of SA algorithms was not recorded since they were finished within a second. For each condition, 100 replications were generated and the results were given in Table 5. It is observed that the branch-and-bound algorithm could solve problems of up to 24 jobs in a reasonable amount of time. However, the number of nodes and execution time grow exponentially as the number of jobs increases since the problem is NP-hard. The most time consuming case (*n*, *P*, *τ*, *R*) = (24,0.25,0.5,0.75) took an average execution time of 47.37 seconds. As to the performance of the SA algorithms, the results showed that both of them are quite accurate with a mean percentage error of less than 1% for all the tested cases. Moreover, it seemed that SA_2_ performed better than SA_1_ when the number of jobs is 16; however, the trend is not obvious as the number of jobs becomes larger. Thus, we would study the performance of the algorithms for large job-size problems in the last part of the experiments.

We tested two job sizes, that is, *n* = 100 and 200, in the last part of the experiments. We randomly generated 100 instances for each situation and we reported the results in Table 6. The mean and standard deviation of the relative deviation percentage (RDP) were given. The RDP of the solution produced by a simulated annealing algorithm is calculated as

(13) $$\begin{array}{c} \frac{\left(V_{i}-\min\left\{V_{1},V_{2}\right\}\right)}{\min\left\{V_{1},V_{2}\right\}}\times 100\% \end{array}$$

for *i* = 1, 2, where *V*_*i*_ is the value of the weighted combination of the total completion time and the total tardiness of the jobs from the first agent generated by the *i*th simulated annealing algorithm. The mean and standard deviation of the execution time were also reported. In addition, we recorded the number of times (*n*_*T*_) it yields the minimum value. It was seen that both algorithms are very fast since the mean execution time is less than 1 second for all the tested cases. However, the first SA algorithm performs better than the second one in terms of the RDP values and the number of times it yields the minimum value. Thus, it is recommended as the number of jobs increases.

## 6. Conclusion

In this paper, we considered a two-agent single-machine scheduling problem where the objective is to minimize the weighted combination of the total completion time and the total tardiness of the jobs of the first agent given that no tardy job is allowed for the second agent. We proposed a branch-and-bound algorithm to solve the problem optimally and two simulated annealing algorithms to find near-optimal solutions. The computational experiments showed that the branch-and-bound algorithm could solve problems of up to 24 jobs in a reasonable amount of time. It also showed that the performance of the first SA algorithm is very good, yielding an average error percentage of less than 1% for all the tested cases.

## Figures and Tables

**Table 1:** The performance of the B&B with *n* = 12 and *θ* = 0.5.

*P *	**τ**	*R *	Number of nodes		CPU time	
			Mean	SD	Mean	SD
0.25	0.25	0.25	275.81	182.83	0.01	0.08
		0.50	389.64	327.66	0.00	0.07
		0.75	496.82	774.90	0.00	0.07
	0.50	0.25	378.42	363.44	0.00	0.06
		0.50	504.80	636.00	0.00	0.07
		0.75	473.48	474.64	0.00	0.06
	0.75	0.25	154.05	169.55	0.00	0.04
		0.5	214.68	244.00	0.00	0.05

0.5	0.25	0.25	323.24	228.37	0.00	0.05
		0.50	345.06	332.54	0.01	0.09
		0.75	362.94	492.78	0.00	0.06
	0.50	0.25	105.47	96.16	0.00	0.03
		0.50	216.62	246.72	0.01	0.08
		0.75	293.75	285.05	0.00	0.06
	0.75	0.25	23.18	35.97	0.00	0.02
		0.5	67.08	105.95	0.00	0.03

0.75	0.25	0.25	115.18	96.11	0.00	0.05
		0.50	160.90	123.03	0.00	0.05
		0.75	104.57	169.79	0.00	0.04
	0.50	0.25	3.95	25.52	0.00	0.03
		0.50	23.55	50.71	0.00	0.02
		0.75	66.48	78.98	0.00	0.03
	0.75	0.25	4.82	20.06	0.00	0.02
		0.5	5.98	29.08	0.00	0.02

**Table 2:** ANOVA table for the number of nodes with *n* = 12  and  *θ* = 0.5.

Source	SS	DF	MS	*F *	*P* value
*P *	2	36236586	18118293	189.52	0.00
(*τ*, *R*)	7	19531459	2790208	29.19	0.00
Error	2390	2.28*E* + 08	95598		

Total	2399	2.84*E* + 08			

**Table 3:** The performance of the B&B with *n* = 12 and *P* = 0.5.

**θ**	**τ**	*R *	Number of nodes		CPU time	
			Mean	SD	Mean	SD
0.25	0.25	0.25	246.56	174.35	0.00	0.05
		0.50	367.78	604.59	0.00	0.07
		0.75	359.06	396.26	0.00	0.05
	0.50	0.25	93.20	61.04	0.00	0.04
		0.50	226.18	264.67	0.00	0.05
		0.75	285.38	297.02	0.00	0.05
	0.75	0.25	25.01	41.37	0.00	0.04
		0.5	44.52	55.99	0.00	0.03

0.5	0.25	0.25	323.24	228.37	0.00	0.05
		0.50	345.06	332.54	0.01	0.09
		0.75	362.94	492.78	0.00	0.06
	0.50	0.25	105.47	96.16	0.00	0.03
		0.50	216.62	246.72	0.01	0.08
		0.75	293.75	285.05	0.00	0.06
	0.75	0.25	23.18	35.97	0.00	0.02
		0.5	67.08	105.95	0.00	0.03

0.75	0.25	0.25	324.93	326.67	0.01	0.10
		0.50	346.91	294.66	0.00	0.06
		0.75	354.06	449.82	0.00	0.06
	0.50	0.25	122.77	114.42	0.00	0.04
		0.50	215.55	217.34	0.00	0.04
		0.75	341.23	301.91	0.00	0.06
	0.75	0.25	26.92	46.26	0.00	0.02
		0.5	46.97	66.25	0.00	0.03

**Table 4:** ANOVA table for the number of nodes with *n* = 12  and *P* = 0.5.

Source	SS	DF	MS	*F *	*P* value
**θ**	2	113054	56527	0.74	0.48
(**τ*, R*)	7	38742472	5534639	72.26	0.00
Error	2390	1.83*E* + 08	76593		

Total	2399	2.22*E* + 08			

**Table 5:** The performance of the B&B and SA algorithms (*θ* = 0.5).

*n *	*P *	**τ**	*R *	Branch-and-bound algorithm				SA_1_		SA_2_	
				Number of nodes		CPU time		Error percentages		Error percentages	
				Mean	SD	Mean	SD	Mean	SD	Mean	SD
16	0.25	0.25	0.25	1585.08	1474.52	0.02	0.02	0.15	0.91	0.11	0.73
			0.50	2095.26	2732.66	0.02	0.03	0.15	0.50	0.14	0.59
			0.75	2222.28	4286.49	0.02	0.04	0.13	0.61	0.14	0.58
		0.50	0.25	2065.72	2321.27	0.02	0.02	0.10	0.59	0.09	0.54
			0.50	4513.85	6357.05	0.04	0.05	0.05	0.32	0.17	0.66
			0.75	8619.26	21890.46	0.07	0.18	0.11	0.31	0.19	0.55
	0.5	0.25	0.25	1393.40	1012.04	0.01	0.01	0.27	1.83	0.15	1.36
			0.50	1705.39	1475.57	0.02	0.02	0.44	2.34	0.45	2.10
			0.75	2492.71	3162.69	0.02	0.03	0.40	1.75	0.20	1.12
		0.50	0.25	708.37	1012.71	0.01	0.01	0.00	0.00	0.00	0.00
			0.50	2342.18	6513.62	0.02	0.05	0.23	0.96	0.31	1.29
			0.75	4389.43	6513.39	0.03	0.05	0.14	0.88	0.17	0.91

20	0.25	0.25	0.25	6440.11	8778.62	0.10	0.13	0.14	0.69	0.21	0.83
			0.50	8888.21	12532.10	0.12	0.16	0.44	1.19	0.29	0.93
			0.75	37910.61	169197.47	0.52	2.44	0.17	0.59	0.29	0.85
		0.50	0.25	31509.93	119592.04	0.41	1.69	0.20	0.72	0.24	0.79
			0.50	77321.28	252992.05	0.98	3.45	0.19	0.73	0.22	0.72
			0.75	107661.85	496233.51	1.33	6.28	0.14	0.41	0.19	0.72
	0.5	0.25	0.25	6080.79	7331.26	0.09	0.10	0.24	1.54	0.15	1.30
			0.50	12825.16	17350.45	0.16	0.21	1.05	3.59	0.96	3.16
			0.75	30411.23	83195.05	0.37	1.01	0.58	2.15	0.53	2.02
		0.50	0.25	3189.29	5854.18	0.04	0.06	0.13	0.93	0.20	1.07
			0.50	20391.67	49137.99	0.22	0.55	0.57	1.76	0.59	1.85
			0.75	86350.21	282470.70	0.95	3.22	0.42	1.19	0.67	1.47

24	0.25	0.25	0.25	28131.45	39702.43	0.59	8.36	0.26	0.77	0.24	0.73
			0.50	100685.98	354838.97	2.15	84.59	0.31	0.84	0.21	0.68
			0.75	281777.41	669598.75	5.79	131.44	0.21	0.62	0.19	0.59
		0.50	0.25	223096.41	1334703.63	4.35	265.11	0.27	0.69	0.25	0.67
			0.50	707912.81	1682584.50	13.42	327.85	0.31	0.64	0.26	0.47
			0.75	2069551.88	4812524.50	47.37	1083.82	0.26	0.50	0.26	0.60
	0.5	0.25	0.25	28694.70	51288.52	0.57	10.11	0.46	2.34	0.80	2.93
			0.50	130640.24	489352.94	2.26	79.84	0.20	1.00	0.44	2.22
			0.75	259434.69	552024.63	4.58	97.95	0.29	1.36	0.86	2.42
		0.50	0.25	17466.58	29918.38	0.26	4.36	0.15	0.76	0.27	1.11
			0.50	439942.63	1794187.38	6.63	298.29	0.46	1.22	0.71	1.75
			0.75	2000465.50	6470223.50	34.15	1047.58	0.49	1.32	0.97	1.63

**Table 6:** The performance of the SA_1_ and SA_2_ algorithms with *θ* = 0.5.

*n *	*P *	**τ**	*R *	RDP				CPU time				*n*_*T*_	
				SA_1_		SA_2_		SA_1_		SA_2_			
				Mean	SD	Mean	SD	Mean	SD	Mean	SD	SA_1_	SA_2_
100	0.25	0.25	0.25	0.07	1.89	0.19	3.07	0.20	0.99	0.23	1.20	64	36
			0.50	0.06	1.67	0.25	2.91	0.23	1.17	0.24	1.21	71	29
			0.75	0.04	1.11	0.21	2.92	0.24	1.20	0.25	1.30	77	23
		0.50	0.25	0.10	2.12	0.18	2.65	0.25	1.24	0.22	1.09	56	44
			0.50	0.07	1.43	0.21	2.26	0.30	1.43	0.25	1.30	72	28
			0.75	0.07	1.33	0.18	2.76	0.37	1.94	0.28	1.37	56	44
	0.5	0.25	0.25	0.44	11.34	0.39	10.38	0.21	1.08	0.23	1.13	56	44
			0.50	0.06	2.92	1.75	18.84	0.23	1.12	0.24	1.24	88	12
			0.75	0.11	4.28	1.49	14.18	0.25	1.26	0.24	1.20	88	12
		0.50	0.25	0.27	5.74	0.31	6.36	0.26	1.32	0.24	1.23	54	46
			0.50	0.22	5.06	0.72	7.84	0.32	1.58	0.25	1.24	74	26
			0.75	0.12	3.36	0.75	6.99	0.45	2.27	0.28	1.40	79	21

200	0.25	0.25	0.25	0.06	1.50	0.28	3.21	0.52	0.14	0.57	0.20	77	23
			0.50	0.07	1.83	0.51	6.29	0.62	0.25	0.61	0.17	77	23
			0.75	0.09	2.06	0.39	4.86	0.69	0.31	0.64	0.30	70	30
		0.50	0.25	0.04	0.98	0.22	2.11	0.70	0.25	0.60	0.27	78	22
			0.50	0.03	0.90	0.32	2.98	0.90	0.32	0.68	0.19	78	22
			0.75	0.04	1.02	0.31	3.24	1.06	0.56	0.80	2.68	76	24
	0.5	0.25	0.25	0.36	7.17	0.48	7.93	0.51	0.21	0.63	1.37	61	39
			0.50	0.17	5.75	2.10	16.13	0.57	0.23	0.65	1.78	85	15
			0.75	0.11	3.21	1.88	17.17	0.67	0.31	0.69	2.30	83	17
		0.50	0.25	0.22	4.05	0.19	3.05	0.76	1.22	0.68	2.34	57	43
			0.50	0.07	1.87	0.89	8.11	1.07	3.21	0.72	2.60	81	19
			0.75	0.01	0.28	1.15	7.52	1.45	4.38	0.87	3.84	95	5
